# PW02-004 - Autoinflammatory syndromes: a clinical review

**DOI:** 10.1186/1546-0096-11-S1-A144

**Published:** 2013-11-08

**Authors:** CM Biggs, JS Hausmann, S Kim, E Janssen, P Nigrovic, R Fuhlbrigge, R Sundel, F Dedeoglu

**Affiliations:** 1 Program in Rheumatology, Division of Immunology, Boston Children's Hospital, Boston, MA, USA

## Introduction

Autoinflammatory syndromes are a group of rare conditions that cause intermittent episodes of fever and organ system inflammation. The majority of these conditions have been linked to single gene mutations that are involved in the acute inflammatory response. Many of these monogenic disorders, such as Familial Mediterranean Fever (FMF) and Hyper IgD syndrome (HIDS), are more prevalent in certain geographic locations such as Europe and the Mediterranean basin. It is therefore not surprising that the literature describing these conditions has largely originated from these regions. Research on these conditions so far suggests variable presentations due to a variety of modifiers such as genetic polymorphisms and/or environment. There is currently a paucity of studies from mixed populations, such as those found in urban North American centers.

## Objectives

To describe the clinical and laboratory features of children with autoinflammatory syndromes as seen in a large, North American pediatric rheumatology clinic.

## Methods

We conducted a retrospective chart review of children evaluated for periodic fevers from 2002-2012 at the Rheumatology Clinic in Boston Children’s Hospital. Charts were first identified by searching for key words or billing codes related to autoinflammatory syndromes. A manual chart review was then performed to confirm appropriate case identification. Diagnosis of autoinflammatory syndromes was based on expert opinion as well as on genetic testing if available.

## Results

One hundred and seventy-four patients were found by the initial search, and 84 patients were excluded after Periodic Fever, Aphthous Stomatitis, Pharyngitis and Adenitis (PFAPA) was identified as the most likely diagnosis. FMF was identified as the most likely diagnosis in 37 patients. Mutations in the MEFV gene were found in 63% of the 27 patients with suspected FMF who underwent genetic testing; 65% had a single heterozygous mutation, while 35% had either 2 homozygous or 2 heterozygous mutations. Thirty-two FMF patients were treated with colchicine and had subsequent follow up, with 100% showing at least a partial response. None of the patients in the FMF group were diagnosed with amyloidosis during an average of 5.3 years of follow-up. Four patients were diagnosed with TNF Receptor Associated Periodic Syndrome (TRAPS) or TRAPS variant, all carrying the R92Q mutation in the TNFRSF1A gene. HIDS was identified in 4 patients, all with confirmed mutations in the MVK gene. Three patients were diagnosed with Cryopyrin Associated Periodic Syndrome (CAPS), with one patient having an identified mutation in the CIAS1 gene. Thirty-nine patients were diagnosed with an undefined autoinflammatory syndrome. Mutations in genes associated with autoinflammatory syndromes were found in 26% of the 19 patients in the undefined group who underwent genetic testing. Eighty-three percent of the 23 patients in the undefined group who were treated with colchicine had at least a partial response.

## Conclusion

This study demonstrates the genetic and phenotypic diversity seen in a mixed North American population. More research is needed to further characterize the clinical features of autoinflammatory syndromes in this population.

## Disclosure of interest

None declared.

